# Richard Hornabrook's first impressions of kuru and Okapa

**DOI:** 10.1098/rstb.2008.4004

**Published:** 2008-11-27

**Authors:** Annette Beasley

**Affiliations:** 112c Paetawa RoadPeka Peka Beach, RD 1, Waikanae, New Zealand

The following recollections are taken from a series of 14 extended interviews conducted with Richard W. (Dick) Hornabrook between February 1995 and February 1998, as part of a larger ethnographic study of the kuru investigation (see Beasley 2004,2006**).

In late 1962, Dick received a letter from Prof. Robert Walsh, on behalf of the newly formed Papua and New Guinea Medical Research Advisory Committee, inviting him as ‘a British-trained neurologist’ to consider a 3-year appointment to study kuru. Following a brief visit to the kuru region in early 1963, Dick recalled that:

The decision to go to New Guinea was really made…within a very short time of seeing the scenario, and was influenced by the warmth and enthusiasm of the people I had interviewed and the confidence I had acquired through…contact with some of the local people in New Guinea.

My understanding of [the situation] was that this was something that I was going to be able to contribute to and [that] my training was right for that contribution. I did not give a great deal of attention in my mind to solving the problem [because] when training in neurology…we were dealing with lots of neurological diseases for which the answer to the problem was indeterminate; solving the problem was not your motivation at all. I quickly decided that as a neurologist the most useful contribution I could make was to describe the disease.

In fact, [the aim of describing the disease] seemed a great challenge to me when I first went there. It was a challenge because of the local people's mistrust, misgivings….It was difficult because people were suspicious of doctors and I was told that the hospital at Okapa was not to be used by kuru patients for political and sociological reasons. Therefore the initial issue was where do I find these patients? How do I go about examining them? I had to devise a way of learning about who got kuru, how they were different from the population at large and what the features and characteristics of the complaint were.

The Hornabrook family arrived at Okapa on 18 December 1963, expecting their household possessions and supplies to follow shortly after.

We had what we had bought in the way of food in Goroka but…we got torrential rain the next day and the road was washed out, so we were short of food for a couple of days but everybody gave us bits and pieces. We didn't have any beds for the children; they actually slept in the drawers of a chest!

On Boxing Day…the Patrol Officer [provided] about twenty pigs…to be killed and the meat [distributed during] a singsing. We went to the front verandah of the house and the little road at the front was…a mass of people beating drums [and] waving spears with huge decorations and painted emblems on poles harnessed to the top of their heads. Different tribal groups came pouring into Okapa all that day until there were probably ten to fifteen thousand people. No European clothing at all! An exciting sight for somebody who hadn't been there before.

Carleton arrived in the middle of the singsing. I think he walked from the airstrip to the station. This was how he operated over the next few years—he would stay [and] we would have really fascinating and stimulating discussions on all manner of topics including, of course, kuru. Then he would disappear.

[Carleton] said I ought to see something of the real New Guinea and [suggested] I go with him to meet these people across the Lamari River. We were taken by a Jeep to the end of the road at Purosa which was at the foot of an escarpment…rising very steeply and heavily forested. I just went along as a helpless innocent! Carleton recruited the cargo boys and we were off. There was me and him and probably a dozen carriers. No other Europeans at all. This was my first venture really off the road…and we went out of the village [keeping to the path by the garden fences…and] all of a sudden there was a mountain wall in front of us. We were really climbing almost hand over knees to speak. The perspiration dripped from every pore of my skin and I didn't know how I was going to survive! Carleton made no concession and he probably crossed the top of the mountain before I had even got a third of the way up. I knew I wasn't fit, I had been [in Wellington] doing nothing much.

One of the inspiring things I still remember to this day [was as] I was toiling [up that mountain with] my butterfly net in one hand a little group of New Guineans arrived. One of them had an old man with very white hair on his head…perched on his shoulders like a little child. A… cargo boy who spoke a little Pidgin…said, ‘This man he close to die. Take him on to die. Finish!’ That encouraged me to get [back up on my feet] and finish the walk! I caught up with the carriers and Carleton. [The carriers]… having finished the worst of the trail were doing something I later became familiar with—they were doing a sort of war cry. An attack cry where they all simultaneously made a sort of series of hooting grunts. Very eerie and very penetrating. I realized then that I was somewhere that I had never been before. It was [all] very alien [to me].

We came to the village which was one of the most spectacular villages… right on the top of a ridge…[that] fell straight to the Lamari River gorge underneath. It was the most magnificent view. [A few days later] we got down to the riverbank. There I became aware of the next hazard of life which was the bridge over the river—a single series of bamboo stems tied together. Carleton proudly told me there wasn't one piece of manufactured equipment in the construction! [The bridge] was about 60 to 100 feet above the water. It was a hell of a distance across. It was agonizing! I was too frightened to get vertigo—you just had to hang on [while] it was swinging from side to side [with] bits falling off into the water all the time. I did get across and was quite proud of myself going the distance.

[After several more days] Carleton and I parted our ways and I had to face the ordeal of getting across that bridge by myself. He gave me two cargo boys… and they escorted me. I actually was going berserk because I didn't intend to be away all that long. I was away for about ten days and Fay had only been in Okapa a week or ten days before that!

Over the ensuing years, Dick Hornabrook interviewed (with the help of interpreters and patrol officers) and examined one of the largest groups of patients suffering from kuru (figure 1), and gave a clear early description of the neurological pattern of symptoms and signs of the disease. Dick's case histories and scrupulous descriptions of his findings remain a vivid testimony to the tragedy of the Fore people's suffering and loss of life to the disease. Paradoxically up until recently, Dick has remembered his time in Papua New Guinea as the happiest memories of his life. Sadly these memories are now lost to him via illness.

## Figures and Tables

**Figure 1:**
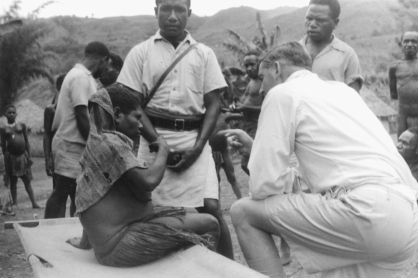
Dick Hornabrook examining, in her village, a patient suffering from kuru.
